# Adequacy of Anesthesia and Pupillometry for Endoscopic Sinus Surgery

**DOI:** 10.3390/jcm10204683

**Published:** 2021-10-13

**Authors:** Michał Jan Stasiowski, Izabela Szumera, Piotr Wardas, Seweryn Król, Jakub Żak, Anna Missir, Aleksandra Pluta, Ewa Niewiadomska, Lech Krawczyk, Przemysław Jałowiecki, Beniamin Oskar Grabarek

**Affiliations:** 1 Chair and Department of Emergency Medicine, Faculty of Medical Sciences in Zabrze, Medical University of Silesia, 40-055 Katowice, Poland; iza_sz@vp.pl (I.S.); seweryn.krol@gmail.com (S.K.); aniami521@interia.pl (A.M.); apluta@autograf.pl (A.P.); lech.kraw@gmail.com (L.K.); olaf@pro.onet.pl (P.J.); 2 Department of Anaesthesiology and Intensive Care, 5th Regional Hospital, 41-200 Sosnowiec, Poland; sernik7@gmail.com; 3 Chair and Clinical Department of Laryngology, Faculty of Medical Sciences in Katowice, Medical University of Silesia, 40-055 Katowice, Poland; pwardas@sum.edu.pl; 4 Department of General, Colorectal and Polytrauma Surgery, Faculty of Health Sciences in Katowice, Medical University of Silesia, 40-555 Katowice, Poland; 5 Department of Neurosurgery, Regional Hospital in Sosnowiec, Faculty of Medical Sciences in Katowice, Medical University of Silesia, 40-055 Katowice, Poland; 6 Department of Epidemiology and Biostatistics, Faculty of Public Health in Bytom, Medical University of Silesia, 40-555 Katowice, Poland; e.j.niewiadomska@gmail.com; 7 Department of Histology, Cytophysiology and Embryology, Faculty of Medicine, University of Technology in Katowice, 41-800 Zabrze, Poland; bgrabarek7@gmail.com

**Keywords:** surgical pleth index, pupillary dilatation reflex, endoscopic sinus surgery, Boezaart bleeding scale, total estimated intraoperative blood loss, condition of the surgical field, total intravenous anesthesia

## Abstract

Inadequate intraoperative analgesia causes the deterioration of the condition of the surgical field (CSF) as a result of hemodynamic instability. Analgesia monitors are used to guide remifentanil) infusion to optimize intraoperative analgesia. The main aim of the current randomized controlled trial was to investigate the potential advantages of intraoperative analgesia monitoring using surgical Pleth index (SPI)- or pupillometry (PRD)-guided remifentanil administration for managing the volume of total intraoperative blood loss (TEIBL), CSF, and length of operation (LOP) in comparison with the standard practice in patients undergoing endoscopic sinus surgery (ESS). The 89 patients in our study were grouped as follows: 30 patients were assigned to the general analgesia (GA) group, 31 patients were assigned to the SPI group, and 28 patients were assigned to the PRD group. The speed of remifentanil infusion was accelerated by 50% when SPI, PRD, or BSS were increased by >15 points, >5%, or >2, respectively, in adjacent groups until their normalization. The SPI group showed significantly lower TEIBL in comparison to the GA group (165.2 ± 100.2 vs. 283.3 ± 193.5 mL; *p* < 0.05) and a higher mean arterial pressure (MAP; 73.9 ± 8 vs. 69.2 ± 6.8 mmHg; *p* < 0.05). In the PRD group, a shorter LOP compared with the GA group was observed (63.1 ± 26.7 min vs. 82.6 ± 33.1 min; *p* < 0.05). It was noted that the PRD group had a lower total remifentanil consumption than the SPI group (1.3 ± 1.4 vs. 1.8 ± 0.9 mg; *p* < 0.05). In ASA I-III patients undergoing ESS, intraoperative monitoring based on state entropy and SPI values can optimize the CSF and reduce TEIBL, whereas monitoring based on state entropy and PRD measurements can optimize the cost effectiveness of anesthetic drugs and the use of the operation room.

## 1. Introduction

Endoscopic sinus surgery (ESS) is currently the surgical treatment of choice for sinonasal disease, and its outcome is dependent on the intraoperative condition of the surgical field (CSF) [1]. Marked intraoperative bleeding during ESS may not only critically hinder the recognition of the anatomical structures of the paranasal sinuses by reducing their visibility but may also extend the length of the operation (LOP) [2].

A wide variety of maneuvers have been proposed to decrease the potential risk of harming the surrounding structures by reducing the amount of total intraoperative blood loss (TEIBL) during ESS performed under general anesthesia. Numerous perioperative regimens, including either premedication or the intraoperative intravenous infusion of beta-blockers [3,4,5] or alpha-2-agonists [2,3], the local infiltration of the mucosa with vasoconstrictors or local anesthetics [5], anesthetic modalities based on different modes of ventilation [6,7], airway management [8], patient positioning in the anti-Trendelenburg position to reduce venous return [9], the controversial preference for total intravenous anesthesia (TIVA) over inhalational anesthesia [10,11,12], and the preference for target-controlled infusions of remifentanil over boluses of other opioid drugs [11], have been reported to improve the CSF and thereby reduce TEIBL.

Surgical maneuvers in the operation field provoke painful afferent stimulation (nociceptive effect), which can induce the release of stress hormones in the absence of sufficient intraoperative analgesia; trigger heart rate and arterial blood pressure increments, resulting in potentially excessive TEIBL; and lead to the administration of recall of awareness tests (ROA attenuates this activity (anti-nociceptive effect)). However, excessively high doses of intravenous intraoperative remifentanil may lead to hemodynamic depression with hazardous bradycardia and hypotension and an increased requirement for intravenous fluids with unwelcome untoward events [13,14,15,16]. Thus, the appropriate adjustment of intraoperative remifentanil infusion constitutes a major challenge in response to the observed fluctuations of the abovementioned hemodynamic parameters accompanied by anesthesiological intuition, since insufficient intraoperative analgesia may not necessarily be reflected in tachycardia and hypertension as anesthetics tend to blunt the hemodynamic response to nociceptive stimulation [17].

Currently, different techniques for monitoring the intraoperative efficacy of analgesia surgical pleth index (SPI), antinociception index (ANI), and pupillary dilatation reflex (PRD) are gaining increasing popularity [18], as in the case of a steady state of depth of anesthesia and muscle relaxation, the only factor influencing hemodynamic changes may be the quality of the intraoperative analgesia. We hypothesize that inadequate intraoperative analgesia may be responsible for excessive TEIBL.

The main aim of the current randomized controlled trial was to investigate the potential advantages of intraoperative analgesia monitoring using SPI- or PRD-guided remifentanil administration for managing the volume of TEIBL, CSF, and LOP in comparison with the standard practice based on the observance of CSF.

## 2. Materials and Methods

### 2.1. Participants

One hundred patients (age, 18–65 years; American Society of Anesthesiologists (ASA) score, I–III) with a history of chronic rhinosinusitis who were scheduled to undergo elective ESS without septoplasty with or without polyposis at the Department of Otolaryngology in Regional Hospital no. 5 in Sosnowiec, Poland, met the inclusion criteria for the present comparative analysis.

The exclusion criteria were as follows: pregnancy, history of allergy to hypnotics, pre-existing cardiovascular diseases (cardiac arrhythmia, general atherosclerosis, poorly controlled hypertension), risk of intraoperative hypotension (low left ventricle ejection fraction < 30%) requiring extensive fluid challenge and the administration of vasoactive drugs that may possibly influence hemodynamic monitoring, low platelet count (<150,000), any pathology in coagulation tests, medication with drugs interfering with clotting.

Randomization was performed by the principal investigator (M.S.) by opening sealed envelopes after each participant provided written informed consent to participate in the study and undergo general anesthesia for ESS. The study protocol was in compliance with the 1964 Helsinki Declaration and was approved by the Local Bioethics Committee at the Medical University of Silesia in Katowice (Poland) on 24 May 2016 (approval number: KNW/0022/KB1/50/16). The study was registered in the Clinical Trials Registry (ID: SilesianMUKOAiIT2, initial release: 9 June 2017).

### 2.2. Anesthesia Technique

Patients fasted for 12 h before ESS. All the patients received 3.75–7.5 mg of midazolam (Midanium, Polfa Warszawa, Poland) in premedication on the day of surgery. Immediately before the surgery, the patients received 10 mL/kg per body weight of Optylite Solution intravenously (i.v.). The patients were preoxygenated with 100% oxygen and intravenously administered fentanyl (Fentanyl WZF; Polfa Warszawa SA, Warsaw, Poland) in a single dose of 2 µg/kg body weight and propofol (Propofol 1% MCT/LCT Fresenius Kabi, Bad Homburg, Germany) in a single dose of 2.5 mg/kg body weight. When the ciliary reflex disappeared, patients in all groups were paralyzed with a standard intravenous dose of rocuronium (Esmeron, Organon, Oss, The Netherlands) at 0.6 mg/kg body weight, and were intubated after approximately 1 min 20 s with a proper-sized cuffed endotracheal tube 7.0–8.5 in the supine position. During the co-induction of general anesthesia, CO_2_ was maintained at 35–37 mmHg. The lungs were ventilated using a low flow (fresh gas flow, 0.7 L/min; oxygen:air ratio, 2:1) and lung-protective strategies with low tidal volumes of 6 mL/kg of ideal body weight.

Throughout the induction and the surgery itself, standard monitoring procedures for vital parameters were used, including non-invasive arterial pressure (NIBP) measurement every 5 min and measurements of systolic arterial pressure (SAP), mean arterial pressure (MAP), diastolic arterial pressure (DAP), heart rate (HR), standard ECG II, arterial blood saturation (SpO_2_), fraction of inspired oxygen in the gas mixture (FiO_2_), and exhaled carbon dioxide concentration (etCO_2_). The depth of anesthesia was monitored using response and state entropy (RE, SE) and muscle relaxation was monitored using the E-NMT module in all patients, despite their group allocation. Surgical pleth index (SPI) measurements using a Carescape Monitor (B650, GE Healthcare, Helsinki, Finland) in patients allocated to the SPI group only and PRD measurements using NeuroLight Algiscan (version 1.15 A5, Marseille, France) in patients allocated to the PRD group only were performed accordingly.

The patients were then anesthetized using the total intravenous anesthesia (TIVA) technique. Propofol and remifentanil were administered in a constant intravenous (Remifentanil, Ultiva, GlaxoSmithKline Export Ltd., Middlesex, UK) infusion to separate venous access. An extra dose of rocuronium was always administered when train of poor (TOF). The depth of anesthesia was maintained using propofol at a target SE value of around 40, since the depth of general anesthesia influences patients’ reactions to painful stimuli.

After the end of the ESS, in order to avoid unacceptable postoperative pain perception a standard dose of non-steroidal anti-inflammatory drug was administered intravenously according to the guidelines and individual patients’ conditions [7]. At the same time, infusions of remifentanil and propofol were stopped and data recording was ceased. When the TOF was >3 and the SE was >88, a standard dose of 0.02 mg/kg of body weight of atropine and reversal agent (Neostigmine Metil Sulfat, Plantigmin Ampul, Polifarma, Istanbul, Turkey) was administered and patients were extubated successfully on the operating table in the operating theatre. After their emergence from TIVA (defined as 9–10 points on the Aldrette Scale), the patients were transferred to the postanesthesia care unit (PACU), where they were observed for at least 1 h.

TEIBL measurements for each patient were performed by the same researcher (I.S.). The blood suction canisters, surgical pads, and irrigation totals were examined to calculate the final blood loss, similar to the technique adopted by DeMaria S et al. [3] All BBS and CSF scores were assessed by the same operator (P.W) without a second blinded observer. Although the use of these methods for estimating TEIBL, BBS, and CSF may not be exact, the measurements were obtained using consistent protocols in all patient groups.

#### 2.2.1. Stage 1

Upon their admission to the operating theatre, a sensor of entropy EEG (RE, SE) was affixed onto the patients’ foreheads according to the manufacturer’s suggestions, a pulse oximeter was attached to their finger contralateral to that used for venous access, an NIBP cuff was attached to their left arm, and the first values were recorded.

#### 2.2.2. Stage 2

The patients were positioned at 15° in the reverse Trendelenburg position to reduce venous congestion. After the induction of TIVA, initial infusion of remifentanil 0.25 µg/kg body weight/minute was started. Randomization was performed simultaneously.

#### 2.2.3. Stage 3 Intraoperatively

On the basis of group allocation, remifentanil infusion was adjusted alongside the maintenance of TIVA as described below.

#### 2.2.4. SPI Group

The SPI value was measured online and recorded with a sampling frequency of 1 min. Each time the SPI value was >15 in comparison with the mean SPI value during stage 2, the remifentanil infusion speed was increased by 50% in the sequence 0.25/0.375/0.5/0.675/etc. µg/kg body weight/min. When the MAP was <65 mmHg, an extra fluid challenge of 5 mL/kg body weight was administered.

Alternatively, to avoid unnecessary hemodynamic complications in cases with HR < 45/min, MAP < 65 mmHg with no reaction to fluid challenge, BBS < 2, and SPI < baseline value, the remifentanil infusion rate was decreased by 50% alongside the administration of a single 10 mg dose of ephedrine (Ephedrinum Hydrochloricum WZF, 25 mg/mL, Polfa Warszawa S.A, Warsaw, Poland).

#### 2.2.5. PRD Group

The PRD was measured and recorded with a sampling frequency of 15 min. Each time the PRD value was >5%, the remifentanil infusion speed was increased by 50% in the sequence 0.25/0.375/0.5/0.675/etc. µg/kg body weight/min. When MAP was <65 mmHg, an extra fluid challenge of 5 mL/kg body weight was administered.

Additionally, each time the BBS value was >2, extra PRD measurements were conducted.

Alternatively, to avoid unnecessary hemodynamic complications in cases with HR < 45/min, MAP < 65 mmHg with no reaction to fluid challenge, BBS < 2, and PRD < 5%, the remifentanil infusion rate was decreased by 50% with the administration of a single 10 mg dose of ephedrine.

#### 2.2.6. GA Group

BBS values were measured and recorded with a sampling frequency of 5 min. Each time the BBS value was >2, the remifentanil infusion speed was increased by 50% in the sequence 0.25/0.375/0.5/0.675/etc. µg/kg body weight/min.

Alternatively, despite the group allocation, to avoid unwelcome bradycardia and hypotension, in cases with HR < 45/min or MAP < 65 mmHg with no reaction to fluid challenge (iv bolus of 5 mL/kg of body weight of Optylite solution), and BBS < 2 the infusion rate of remifentanil was reduced to 50% of the initial infusion speed alongside the administration of a single 10 mg dose of ephedrine.

**Figure 1:**
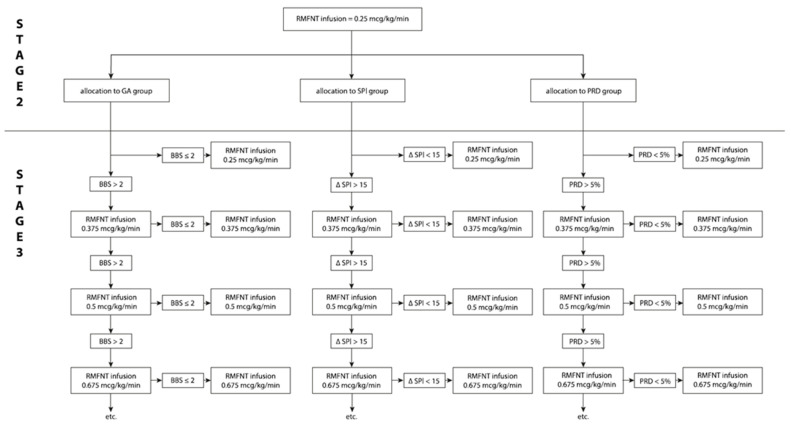
The sequence of events relating to the adjustment of remifentanil infusion according to group allocation (SPI, PRD and GA). RMFNT, remifentanil; GA group, general anesthesia group; SPI group, surgical pleth index group; PRD group, pupillary dilatation reflex group; BBS, Boezaart bleeding scale.

The sequence of events related to the adjustment of remifentanil infusion according to the group allocation (SPI, PRD, and GA) values is shown on the graph (Figure 1).

### 2.3. Technique of FESS and Surgical Considerations

After the induction of TIVA, pledgets impregnated with topical vasoconstrictor were administered to the nasal cavity for the duration of stage 2. All ESS procedures were performed by the same specialist in otolaryngology with over 10 years of experience in this field (P.W), who performed either sphenoethmoidectomy with medial antrostomy (the most severe procedure), total ethmoidectomy with medial antrostomy, anterior ethmoidectomy with medial antrostomy, or isolated antrostomy (the least severe procedure) using powered and manual instrumentation. When the patients showed bleeding that did not respond to topical vasoconstriction, bipolar cautery was administered and standard nasal dressings were placed within the middle meatus, as required.

CSF estimations were performed every 5 min by the operator on the basis of the Boezaart bleeding score (BBS) (0, no bleeding (cadaveric condition); 1, slight bleeding, no suctioning required; 2, slight bleeding, occasional suctioning required; 3, slight bleeding, frequent suctioning required and bleeding threatens the surgical field a few seconds after suction is removed; 4, moderate bleeding, frequent suctioning required and bleeding threatens the surgical field directly after suction is removed; 5, severe bleeding, constant suctioning required and bleeding appears faster than can be removed by suction, with the surgical field being severely threatened and surgery usually not possible) [16].

The time of surgery was counted from the moment of first insertion into the nasal cavity until the last withdrawal of the endoscope from the nasal cavity.

### 2.4. Statistical Analysis

The sample size was estimated at 100 considering the total number of surgeries performed (average, *n* = 135 per 1.5 years), a confidence level of 95%, and a margin of error of 5%. Statistical calculations were performed using MS Excel, STATISTICA 13, and Stat Soft Poland. The measured data were characterized using the mean and standard deviation (X ± SD), as well as median with interquartile range (M (IQR)). The normality of distribution was assessed using the Shapiro–Wilk W test. The significance of the differences between means was tested using a one-way ANOVA, and for skewed distributions the compatibility in groups was examined using the Kruskal–Wallis test. To explore differences between multiple groups, the appropriate post hoc tests were used. For nominal data, we used percentages. The relationships between nominal variables were verified by the χ2 test of independence. Statistical significance was set at *p* < 0.05.

## 3. Results

**Figure 2:**
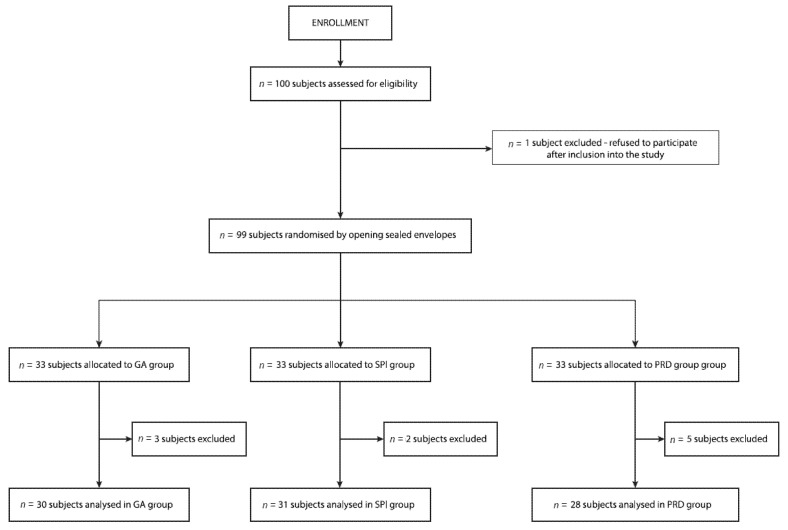
Randomization graph. GA group, general anesthesia group; SPI group, surgical pleth index group; PRD group, pupillary dilatation reflex group.

The current analysis included 89 patients out of the 100 recruited for the study. One patient withdrew their previous consent for participation after being included in the study, while three, two, and five patients allocated to the GA, SPI, and PRD groups were excluded from the final analysis for the following reasons: the presence of an irregular heart rhythm impairing SPI monitoring in one patient, the necessity of the administration of vasoactive drugs due to hypotension necessitating reductions in the speed of REMIFENTANIL infusion and fluid challenge in two subjects, uncontrolled surgical hemorrhage with blood loss over 700 mL in four subjects, and technical problems with PRD measurement connected to the vaporization of the optical system of the pupillometer in three subjects (Figure 2).

The 89 patients included 33 (37.1%) women and 56 (62.9%) men, who were grouped as follows: GA, 30 patients (33.7%); SPI, 31 patients (34.8%); and PRD, 28 patients (31.5%). The detailed patient characteristics are shown in Table 1. No significant intergroup differences were observed in terms of age, gender, height, weight, or BMI.

All operated patients were divided into four categories depending on the severity of the surgery performed: sphenoethmoidectomy with medial antrostomy (the most severe procedure), total ethmoidectomy with medial antrostomy, anterior ethmoidectomy with medial antrostomy, or isolated antrostomy (the least severe procedure). This classification provided the best representation of the difficulty and duration of the procedure, thus allowing the best assessment of the major factors influencing the bleeding. Another criterion of similar importance for the final result was the distinction between single- and double-sided procedures. This system of division allowed for the statistical estimation of homogeneity among the three groups tested (GA, SPI, PRD) with respect to the surgical procedure performed (Table 2).

There were no significant differences in terms of the number of surgical procedures carried out. This allowed us to eliminate the possibility of the unwanted influence of the type of surgical procedure performed on intraoperative bleeding. No statistically significant intergroup differences were observed in the percentages of surgical procedures performed (Table 2).

**Figure 3:**
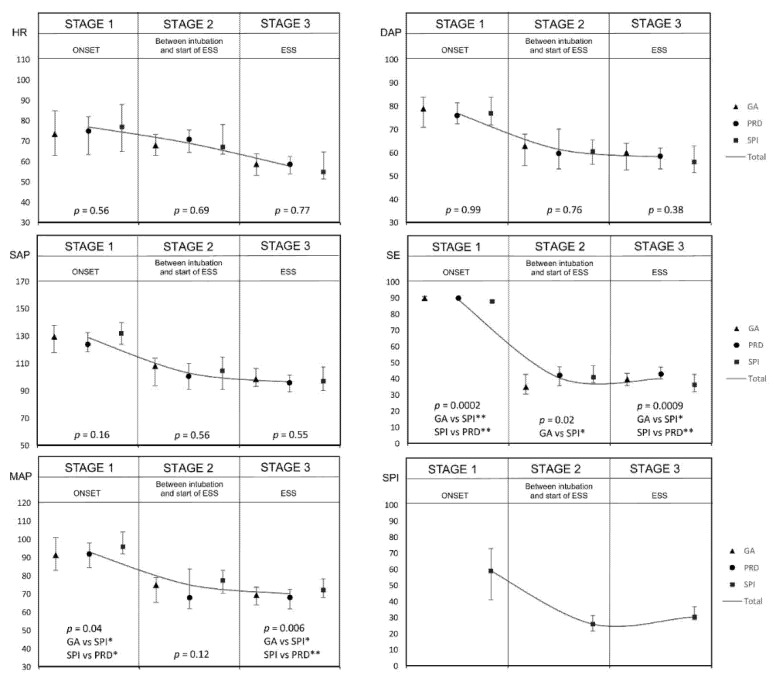
Comparison of the mean values of patient parameters monitored at the same stage between the studied groups (* *p* < 0.05, ** *p* < 0.01). Abbreviations: GA group, general anesthesia group; SPI group, surgical pleth index group; PRD group, pupillary dilatation reflex group; HR, heart rate; SAP, systolic arterial pressure; MAP, mean arterial pressure; DAP, diastolic arterial pressure; SE, state entropy.

**Figure 4:**
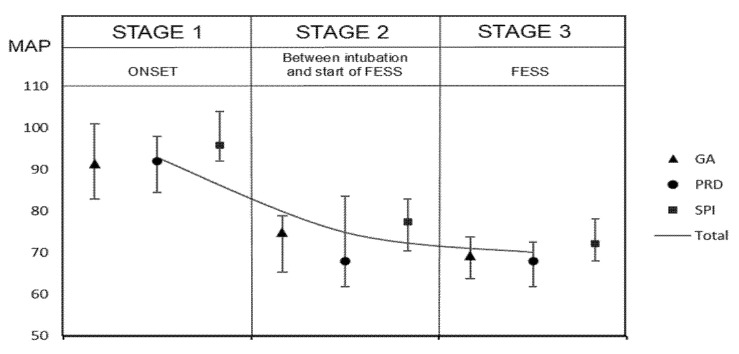
Study outline alongside the differences in MAP values between the study groups. Results are presented as medians (IQR) for quantitative variables. GA group, general anesthesia group; SPI group, surgical pleth index group; PRD group, pupillary dilatation reflex group; MAP, mean arterial pressure; ESS, endoscopic sinus surgery.

Figure 3 presents the mean values of the monitored parameters at the individual stages of the procedure. Significantly higher MAP values were noted in the SPI group (Figure 4).

**Figure 5:**
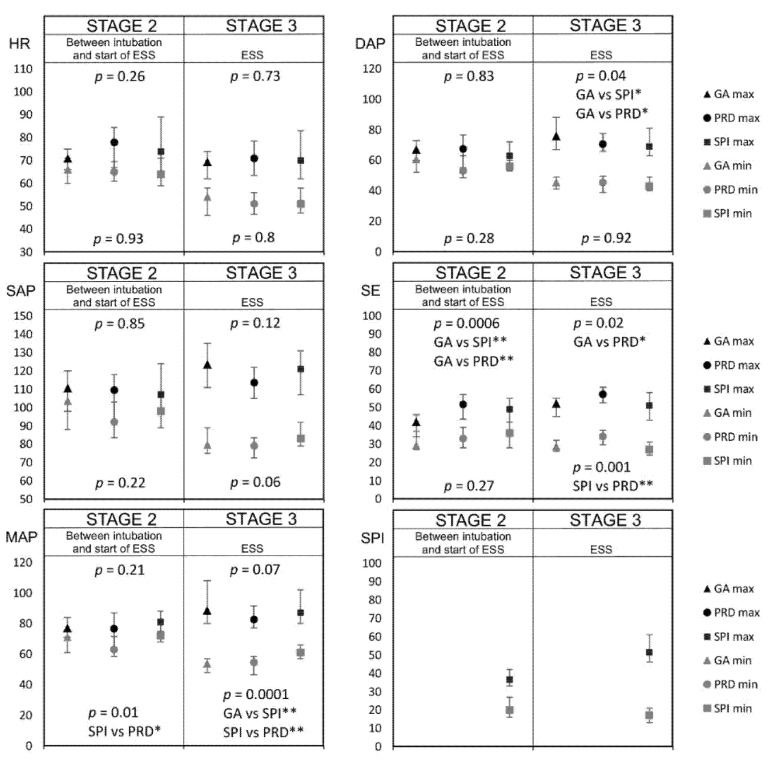
Comparison of the hemodynamic fluctuations in the values of the patient parameters monitored at the same Scheme 0. * *p* < 0.05, ** *p* < 0.01). Abbreviations: GA group, general anesthesia group; SPI group, surgical pleth index group; PRD group, pupillary dilatation reflex group; HR, heart rate; SAP, systolic arterial pressure; MAP, mean arterial pressure; DAP, diastolic arterial pressure; SE, state entropy.

The GA group showed a significantly higher intraoperative blood loss, length of surgery, propofol consumption, and maximum speed of remifentanil infusion, while the SPI group showed a higher total remifentanil consumption and higher minimum and mean speeds of remifentanil infusion (Table 3). A comparison of the minimum and maximum values of the parameters is shown in Figure 5.

## 4. Discussion

Obtaining a “dry” surgical field—assessed, for example, using the BBS—has become a matter of great concern for patients undergoing ESS, as excessive intraoperative bleeding in the surgical field can lead to a number of unwelcome untoward life-threatening complications, such as the disruption of neighboring structures (e.g., the carotid artery, optic nerve, eye globe, or dura matter) as a result of bleeding impairing visibility [1,17,18]. For this study, we decided to use the 5-degree BBS, since it is a more widely used system and because it allowed the simplification of the calculations. Thus, the current comparative prospective study aimed to assess the influence of SPI or PRD monitoring in comparison with BBS monitoring for guiding the speed of remifentanil infusion on the TEIBL and CSF volumes and LOP in patients undergoing ESS under TIVA. The SPI-based control of remifentanil infusion speed during general anesthesia, reflecting a nociception–antinociception balance, was proven to be more effective than control based on hemodynamic changes in reaction to painful intraoperative stimuli [19,20,21]. An increase in the SPI values shown on the monitor (0, no afferent nociceptive stimulation; 100, maximum afferent nociceptive stimulation) after nociceptive afferent stimulation and their return to a baseline level after the acceleration of remifentanil infusion simplified the monitoring of intraoperative titration and made it more reliable because the variations in the SPI values in response to nociceptive stimulation correlated with the remifentanil serum concentration [22,23], thereby reducing the cumulative remifentanil requirement during general anesthesia [24,25]. Moreover, the collection of signals from finger photoplethysmography did not require complex preoperative preparations [26,27].

For PRD, the intraoperative guidance of opioid titration was based on objective measurements of pupil sizes and pupillary reflexes using portable infrared pupillometers reflecting the reactions of the human pupils to drugs and nociceptive stimulation. During the session, the patient’s eye was flooded with infrared light and the reflected images were measured on an infrared sensor, with the variations in pupil size, alongside the pupillary light reflex and pupillary reflex dilatation, being calculated by the instrument and displayed on a screen immediately after each time-stamped measurement. The monitoring of the quality of intraoperative analgesia using pupillometry has proven to be useful in the management of pain because it allows for the assessment of the effect of opioids and the titration of anesthetics [28,29], and it can reduce the intensity of acute postoperative pain perception and analgesic consumption in the first 12 h after major gynecological surgery [30,31].

Karkanevatos et al. [32] analyzed the size and light reflex of the pupils during FESS in the group of 20 patients under GA. In all patients, mysosis and light reflex were noticed. The authors indicated that monitoring pupillary size and reflexes during FESS can be difficult due to the occurrence of myosis during GA [32].

In turn, Sabourdin et al. [33] performed a single-blinded, prospective, parallel-arm randomized study. The study aimed to assess the impact of intraoperative pupillometry monitoring on perioperative opioid consumption during gynecologic surgery. It was observed that remifentanil consumption was significantly decreased in the pupillometry group in comparison to the control group. Nonetheless, no adverse events related with pupillometry were observed. The authors showed reduced intraoperative remifentanil consumption and postoperative morphine requirements in women who underwent intraoperative pupillometry monitoring [33].

In our study, despite the group allocation, we employed state entropy (SE) to achieve similar conditions for all subjects in terms of central nervous system suppression, which was achieved using propofol infusion. The depth of anesthesia markedly influences the intraoperative pain perception. SE within a range of 40–60 is known to reflect the proper surgical depth of anesthesia, and the target SE in our study was set at around 40 (mean SE: 40.1 ± 6.7 despite group allocation) to provide comparable conditions for all subjects in the study. Although the mean SE in the PRD group was significantly higher than that in the SPI and GA groups (43.7 ± 4.9 vs. 37.4 ± 7.8 vs. 39.4 ± 5.3, respectively), the three different anesthetic regimens could be reliably compared, since such differences showed little clinical relevance and because the majority of studies in the recent literature pay no attention to the depth of anesthesia in individual subjects undergoing ESS.

We hypothesized that intraoperative surgical manipulations lead to afferent nociceptive stimulation, resulting in increased blood pressure and heart rate and eventually excessive bleeding impairing the CSF, the intensity of which would vary depending on the stage of ESS (e.g., uncinectomy, opening the antrostomy), with varied hemodynamic changes occurring according to the intensity of the surgical maneuvers performed.

In the current study, the final analysis showed a statistically significant difference between the SPI and GA groups in terms of TEIBL (*p* < 0.05) and no significant difference from the PRD group. In our study, the online observation of SPI value changes alongside the online adjustment of remifentanil infusion speed in the case of a delta SPI increase >15 points over the baseline level supposedly resulted in the effective suppression of afferent nociceptive stimulation before the increase in heart rate and systolic blood pressure resulted in excessive blood loss. In contrast to SPI monitoring, PRD assessments could not have been performed online due to the manufacturer’s indications. Therefore, nociceptive afferent stimulation in the PRD group led to some hemodynamic decompensation, resulting in increased blood loss. However, this was subsequently suppressed by accelerating the FMFNT infusion after PRD measurement (sensitivity > 5%), and no statistically significant difference was noted between the PRD and GA groups.

According to the literature, the values of TEIBL and CSF vary according to the anesthetic regimen used. Lee et al. [21] compared intraoperative remifentanil infusion with dexmedetomidine target infusion guided by the observance of hemodynamic changes and achieved slightly worse mean values of TEIBL (around 184 and 214 mL, respectively) than those observed in the SPI group in the current study. Nair et al. [16] observed slightly better mean TEIBL values in their study among patients receiving beta-blockers in premedication (around 150 mL) as compared with the SPI group in the current study. The administration of beta-blockers possibly reduced TEIBL via the vasoconstriction of the mucous membrane arterioles and precapillary sphincters as a result of the unopposed alpha-adrenergic effects of endogenous catecholamines, in addition to the possible blunting of the sinus node’s acceleration of the heart rate in reaction to nociceptive afferent stimulation. Although the results in the current study were not as promising as those obtained by Ping-Hung Shen et al. [29], who evaluated TEIBL after an infusion of esmolol (constituting a subsequent cost markedly impairing the economic frugality of the anesthesia administered) in comparison with a saline group (69.5 ± 13.1 vs. 101.5 ± 14.8 mL,, respectively, *p* = 0.111), the authors underlined the limited application of their anesthetic regimen to patients with ASA I-II status due to the subsequent risk of myocardial depression, whereas the current analysis also included 14 ASA III patients.

Very impressive TEIBL values were also achieved by Cardesín A et al. [34], with patients receiving clonidine showing lower TEIBL values (83.6 ± 84 mL) than those receiving opioid derivatives (136.7 ± 98.6 mL), but due to the exclusion criteria in their study including any contraindication for using clonidine (a history of coronary artery disease, heart rhythm disorders, blood pressure < 60 mmHg, moderate to severe heart failure, signs of hypovolemia, and regular treatment with alpha- or beta-adrenergic blockers or calcium antagonists), only 11 subjects met the criteria for premedication with clonidine in their study compared with 31 patients in our SPI group.

The overall cumulative dose of remifentanil and propofol given failed to reach statistical significance in the SPI guidance and optimized the intraoperative use of TIVA without sparring effect and influence on LOP. In the PRD group, the LOP was significantly shorter (63.1 ± 26.7 min) than that in the control group (82.6 ± 33.1 min). The introduction of PRD-guided remifentanil administration in our study resulted in a shorter LOP than that in the study conducted by Lee J et al. [21], who compared intraoperative remifentanil (LOP, 78.81 ± 25.12 min) and dexmedetomidine (82.47 ± 24.40 min) target infusion guided by the observance of hemodynamic changes and obtained values comparable to those found in the control group in the current study with a similar anesthetic regimen. In our study, the lowest TEIBL in the SPI group was achieved at the cost of the highest total remifentanil consumption (1.8 ± 0.9 mg) in comparison with the PRD group (1.3 ± 1.4 mg).

Interestingly, the novel approach to the intraoperative monitoring of analgesia during ESS adopted in the current study appeared to be safer for patients in the SPI group compared with the control group in view of the fact that profound hypotension was reported to lead to severe postoperative complications, such as the impairment of cerebrovascular autoregulation and heart function, multi-organ failure, personality changes, and death [18,31,35,36,37]. The mean MAP values reported in the SPI group were higher than those in the control group (73.9 ± 8 vs. 69.2 ± 6.8 mmHg, respectively; *p* < 0.05), and they were also far from the definition of controlled hypotension, whereas the CSF and TEIBL values were surprisingly even more acceptable. Blood pressure reductions, which constitute an independent risk factor for the occurrence of myocardial ischemia in high-risk patients, were not as low in the SPI group as in the PRD and control groups (min MAP: 61.6 ± 7.5 vs. 53.6 ± 7.6 vs. 53.1 ± 9.5 mmHg, respectively; *p* < 0.001). Thus, the anesthetic regimen used in the current study consisting of SPI-guided remifentanil infusion provided patient safety with a satisfactory TEIBL.

In the study of Nair S et al. [16], the authors’ crucial conclusion was that, during ESS, the anesthetic regimen should be directed towards the maintenance of a low heart rate because they observed a correlation between BBS and heart rate, but no correlation with mean arterial blood pressure, in the entire group of patients. Similar observations were made by Ping-Hung Shen et al. [29] and Eberhart LH et al. [5], but they included only ASA I-II patients, as hemodynamic instability, including bradycardia with hypotension, can make surgery hazardous and jeopardize the success of ESS. The abovementioned observations were not proven in our study, since the mean HR was 58.6 ± 7.9 beats/minute despite the group allocation and the intergroup differences did not reach statistical significance, although the mean values for TEIBL, LOP, and the total consumption of anesthetics did.

Our study has several limitations. Although the anesthetic regimen of SPI-guided remifentanil infusion is usually based on the assumption that any SPI value > 50 or intraoperative delta SPI > 10 constitutes an indication of the acceleration of remifentanil infusion [38], we adopted a more liberal protocol (delta SPI >15 as compared with mean SPI value before the start of FESS, stage 2) to avoid potential miscalculations and hazardous uncontrolled hypotension and bradycardia [18,39,40,41,42,43]. We have applied the same methodology in several recently published studies [44,45,46,47]. Furthermore, a stricter protocol could possibly have led to even more impressive intraoperative outcomes at the cost of excluding ASA III patients, limiting the application of these novel techniques, or imposing unnecessary risks of perioperative complications [47]. Moreover, intraoperative PRD measurements require timing and accurate cooperation with the operator, since the operation field lies close to the area of measurement, and this may constitute a major obstacle for some anesthesiologists. The fact that each PRD measurement lasted a minute might have biased the time duration of ESS in the PRD group, despite the fact that the anesthesiologists tried to adjust the PRD measurement timing between the stages of operator’s separate manipulations in the operation field. Due to the word count limit, we have decided to write an additional report that will present correlations between the changes in SPI or PRD values at all stages of the study with the SAP, MAP, DAP, HR, and BBS values.

## 5. Conclusions

Our results are similar to observations made by other researchers. This procedure may impose either a substantial cost or constitute a risk of potential cardiovascular complications while excluding numerous ASA III patients, whose population is growing. We recommend the introduction of adequacy of anesthesia (AoA) monitoring based on SE and SPI to optimize the CSF and reduce the total estimated intraoperative blood loss in a wider group of patients, who may otherwise receive either premedication with clonidine or an expensive intravenous infusion of esmolol. The introduction of intraoperative monitoring based on state entropy and PRD measurements will help us to optimize resources related to the cost-effectiveness of drugs and the use of an operation room.

## Figures and Tables

**Table 1:** Anthropometric data from the GA, SPI, and PRD groups.

**Metrics**		**Total**	**GA Group**	**SPI Group**	**PRD Group**	***p*** **-Value**
		***n*** **=** **89 (100%)**	***n*** **=** **30 (33.7%)**	***n*** **=** **31 (34.8%)**	***n*** **=** **28 (31.5%)**	
age	[years]	50.2 ± 14.6 51 (27)	49 ± 15.4 51 (26)	47.7 ± 13.9 46 (21)	54.1 ± 14.4 59.5 (23)	0.22
gender	male	56 (62.9)	20 (66.7)	18 (58.1)	18 (64.3)	0.77
	female	33 (37.1)	10 (33.3)	13 (41.9)	10 (35.7)	
**Anthropometric Data**		**Total**	**GA Group**	**SPI Group**	**PRD Group**	***p*** **-Value**
		***n*** **= 89 (100%)**	***n*** **= 30 (33.7%)**	***n*** **= 31 (34.8%)**	***n*** **= 28 (31.5%)**	
height	[cm]	171.4 ± 9 171 (12)	173.5 ± 9.3 175 (9)	170.9 ± 8.7 170 (12)	169.6 ± 9.1 170 (12)	0.26
weight	[kg]	78.2 ± 14.6 79 (20)	80.3 ± 11.5 81 (18)	78.3 ± 14.7 79 (25)	75.9 ± 17.3 74 (23.5)	0.52
BMI	[kg/m^2^]	26.5 ± 3.9 26.6 (5.2)	26.8 ± 3.9 26.9 (4.4)	26.6 ± 3.6 27 (4.6)	26.1 ± 4.2 25.2 (5.2)	0.82
BMI	norm	29 (32.6)	9 (30)	7 (22.6)	13 (46.4)	0.38
	overweight	43 (48.3)	15 (50)	18 (58.1)	10 (35.7)	
	obesity	17 (19.1)	6 (20)	6 (19.4)	5 (17.9)	
ASA	I	25 (29.8)	9 (31)	11 (37.9)	5 (19.2)	0.31
	II	45 (53.6)	18 (62.1)	14 (48.3)	13 (50)	0.52
	III	14 (16.7)	2 (6.9)	4 (13.8)	8 (30.8)	0.05
LM CT scale mean	LM < 12	50 (56.2)	12 (40)	19 (61.3)	19 (67.9)	0.08
	LM ≥ 12	39 (43.8)	18 (60)	12 (38.7)	9 (32.1)	
primary EES/revision		74 (84.1)	25 (86.2)	26 (83.9)	23 (82.1)	0.91
samter’s triad		7 (8.3)	5 (17.2)	0 (0)	2 (7.7)	0.05
asthma		17 (20.2)	10 (34.5)	4 (13.8)	3 (11.5)	0.06
arterial hypertension		31 (36.9)	10 (34.5)	10 (34.5)	11 (42.3)	0.79
coronary artery disease		7 (8.3)	2 (6.9)	1 (3.4)	4 (15.4)	0.27

GA group, general anesthesia group; SPI group, surgical pleth index group; PRD group, pupillary dilatation reflex group; ESS, endoscopic sinus surgery; BMI, body mass index; ASA, American Society of Anesthesiologists.

**Table 2:** Comparison of patients according to the surgical procedures performed between studied groups.

Surgery	Total	GA Group	SPI Group	PRD Group	*p*-Value
	*n* = 89 (100%)	*n* = 30 (33.7%)	*n* = 31 (34.8%)	*n* = 28 (31.5%)	
unilateral/bilateral	26 (29.2)	6 (20.0)	10 (32.3)	10 (35.7)	0.42
	62 (70.8)	23 (77.0)	21 (67.7)	18 (64.3)	
antrosthomy with sfenoetmiodectomy	41 (46.6)	17 (58.6)	12 (38.7)	12 (42.9)	0.27
antrosthomy with total etmoidectomy	19 (21.6)	6 (20.7)	6 (19.4)	7 (25)	0.86
antrosthomy with anterior etmoidectomy	28 (31.8)	6 (20.7)	13 (41.9)	9 (32.1)	0.21
isolated anthrostomy	-	-	-	-	-

GA group, general anesthesia group; SPI group, surgical pleth index group; PRD group, pupillary dilatation reflex group.

**Table 3:** Intraoperative parameters in patients according to their allocation to the studied groups.

Intraoperative Parameters	Total	GA Group	SPI Group	PRD Group	*p*-Value
	*n* = 89 (100%)	*n* = 30 (33.7%)	*n* = 31 (34.8%)	*n* = 28 (31.5%)	
total intraoperative blood loss tibl (mL)	207.5 ± 154.3 170 (200)	283.3 ± 193.5 220 (300)	165.2 ± 100.2 150 (150)	173.1 ± 128.6 135 (189)	0.04 *p* < 0.05 ga vs. spi *
length of operation lop (min)	74.1 ± 32.3 73 (35)	82.6 ± 33.1 85 (40)	75.8 ± 34.2 70 (39)	63.1 ± 26.7 65.5 (35)	0.05 ga vs. prd *
total propofol consumption (mg)	666 ± 269.5 620 (340)	762.3 ± 273.2 750 (350)	665.8 ± 237.8 650 (400)	562.9 ± 269.1 520 (285)	0.008 *p* < 0.01 ga vs. prd **
total RMFNT consumption (mg)	1.6 ± 1.2 1.5 (1.1)	1.7 ± 1.1 1.5 (1)	1.8 ± 0.9 1.8 (0.9)	1.3 ± 1.4 1 (0.7)	0.005 *p* < 0.01 spi vs. prd **
max speed of RMFNT infusion (mcg/kg/min)	0.38 ± 0.16 0.38 (0.25)	0.42 ± 0.21 0.38 (0.25)	0.4 ± 0.12 0.38 (0.25)	0.33 ± 0.15 0.25 (0.13)	0.02 *p* < 0.05 ga vs. prd *
min speed of RMFNT infusion (mcg/kg/min)	0.22 ± 0.06 0.25 (0.13)	0.21 ± 0.06 0.25 (0.13)	0.25 ± 0.05 0.25 (0)	0.21 ± 0.07 0.25 (0.13)	0.03 *p* < 0.05
mean speed of RMFNT infusion (mcg/kg/min)	0.31 ± 0.12 0.28 (0.12)	0.32 ± 0.14 0.28 (0.16)	0.33 ± 0.09 0.34 (0.16)	0.27 ± 0.12 0.25 (0.1)	0.007 *p* < 0.01 spi vs. prd **
max values of BBS	2.7 ± 0.7 3 (1)	2.9 ± 0.7 3 (1)	2.5 ± 0.6 3 (1)	2.6 ± 0.7 3 (1)	0.2
min values of BBS	1.7 ± 0.5 2 (1)	1.8 ± 0.4 2 (0)	1.7 ± 0.5 2 (1)	1.6 ± 0.5 2 (1)	0.09
mean values of BBS	2 ± 0.4 2.1 (0.3)	2.1 ± 0.5 2.2 (0.4)	1.9 ± 0.5 2 (0.5)	2 ± 0.3 2 (0.2)	0.07
mean time duration of BBS > 2	12.7 ± 15.3 5 (20)	16.3 ± 16.8 15 (30)	11.8 ± 14.9 5 (25)	9.6 ± 13.7 5 (15)	0.23
mean number of incidences of increased value of BBS > 2	1.07 ± 1.1 1 (2)	1.4 ± 1.3 1 (2)	0.9 ± 0.9 1 (1)	0.89 ± 1 1 (1.5)	0.19

Abbreviations: GA group, general anesthesia group; SPI group, surgical pleth index group; PRD group, pupillary dilatation reflex group; BBS, Boezaart bleeding scale; MIN, minimum; MAX, maximum; RMFNT, remifentanil; kg, kilogram; mcg, microgram; min, minute; * *p* < 0.05; ** *p* < 0.05.

## Data Availability

The data used to support the findings of this study are included in the article.
